# A study of the coupling between the digital economy and regional economic resilience: Evidence from China

**DOI:** 10.1371/journal.pone.0296890

**Published:** 2024-01-19

**Authors:** Jingshan Gu, Zongting Liu

**Affiliations:** 1 School of Economics and Management, Weifang Institute of Science and Technology, Weifang, China; 2 School of Management, Shandong University of Technology, Zibo, China; Qufu Normal University, CHINA

## Abstract

The contemporary economic landscape has placed significant emphasis on the digital economy and economic resilience, progressively emerging as pivotal focal points for examining the high-quality development of economic systems. However, there remains to be more research on several critical topics. This includes the characteristics of coordinated development between the digital economy and economic resilience systems and their interdependence. In response, this study formulates a comprehensive evaluative framework for digital economy development and regional economic resilience, grounded in the intrinsic mechanisms of both domains. It conducts a thorough evaluation employing entropy weight-TOPSIS methodology. Additionally, leveraging coupling theory, a coordination model’s coupling degree serves as the foundational framework for scrutinizing the symbiotic advancement of the digital economy and economic resilience, along with their interdependent nature. The research sample comprises data from 31 provinces and municipalities in China (excluding Hong Kong, Macao, and Taiwan) from 2011 to 2020. Spatial autocorrelation and Geodetector methodologies probe the evolutionary traits and driving factors underlying the coordinated developmental relationship between these two systems. The findings indicate an upward trajectory in China’s annual comprehensive development index for digital economy development (from 0.233 to 0.458) and regional economic resilience (from 0.393 to 0.497). The coupling and coordination between the two systems, measured from 0.504 in 2011 to 0.658 in 2020, demonstrate a consistent growth pattern with an average annual increase of 3.01%. These levels exhibit continuous improvement, with comprehensive economic zones manifesting hierarchical results within the coupling range of [0.5, 0.8]. Notably, agglomeration development evinces a pronounced spatial positive correlation, while local Moran scattering points are primarily concentrated in localized migration leaps. Factors such as foreign-funded enterprises’ total import and export volume, online payment capability, and fiber-optic cable length greatly influence the coupling relationship. In contrast, other variables exhibit a lower and more fluctuating degree of weighted impact. This study establishes a foundation for the synergistic and effective development of the digital economy and economic resilience within the Chinese region. Simultaneously, it offers valuable insights for research of related subjects in global contexts.

## 1 Introduction

With the continuous evolution of the Internet, there has been a sustained progression in the capabilities of information technology pertaining to human processing, storage, analysis, and transmission. Digital processing techniques have emerged as they transcend geographical constraints, enabling information components’ swift and efficient dissemination. This surge in capability has generated significant market demand for digital technology. In response, the digital technology domain swiftly expanded into the commercial sphere, heralding the commencement of the digital economy era [1]. Given the influence of the multifaceted pandemic and the intricate international scenario, all nations are now confronted with a new trial in economic development. Economic resilience has emerged as a pivotal determinant, functioning as the linchpin for ensuring robust economic advancement, particularly in adverse environments. The burgeoning digital economy, as an innovative economic paradigm, has infused new vigor into the economic landscape. It has not only facilitated the evolution of the economic framework across various dimensions but also established itself as a vital impetus, empowering economic resilience to confront risks and adapt to shifts and allocations in resources.

From the start of the 21st century, the global economy has observed heightened dynamism, and among the innovative economic forms, the digital economy has exhibited an exponential growth trajectory. As stated in the Digital China Development Report (2022) released by the China Academy of Information and Communication Technology (AICT), China’s digital economy attained a scale of 45.5 trillion yuan in 2021, constituting 39.8% of the nation’s GDP. The establishment of "Digital China" has emerged as a crucial pillar for ensuring the stability of China’s national economy. The rapid growth of China’s digital economy is closely linked to its robust material and technological infrastructure and its vast market resources, which provide the necessary objective conditions. Additionally, China’s economic solid resilience plays a pivotal role by fostering an inclusive and open development model supported by policies that drive digital transformation [2]. Over the past few years, China’s economic system has showcased outstanding resilience when confronted with internal and external shocks. It possesses a stable economic foundation and the capacity to withstand and adapt to risks, ensuring the achievement of high-quality economic development objectives. With ample room for buffering on both the supply and demand sides, China’s economic resilience continues to improve, presenting significant potential for sustainable development. The country boasts advantages across various industrial chains, particularly emerging high-end industries. Furthermore, a conducive environment exists for developing new types of businesses, including information and communication technology, e-commerce, and the Internet economy [3]. As the world’s most promising market, China possesses diverse and abundant market demand and resilience, making it highly attractive for resource factors. Innovative technologies are critical in developing the digital economy, driven by market demand, which fosters innovation, facilitates technological advancements, and stimulates industry growth. Nevertheless, despite the rapid growth of the digital economy and the systematic improvement of economic resilience, the coordination and promotion relationship between these two domains still needs to be clarified. Exploring the development path under a synergistic mode, identifying the evolving characteristics of the stage of synergistic development between China’s digital economy and economic resilience, and examining the geopolitical disparities are all crucial aspects to consider. Addressing these aspects is crucial for keeping China’s economic development on track within the new typical economic environment.

Similarly, on a global scale, the digital economy, as a new economic form, aligns more closely with the characteristics of current economic and social development stages. Moreover, the core connotation of economic resilience provides the necessary support for developing the digital economy. The fluidity of the digital economy, capable of transcending geographical limitations, directly impacts the adaptability and adjustment capabilities within regional economic resilience. Conversely, this influence will also offer support in the process of collision and integration between the digital economy and traditional economy, accelerating the assimilation of different economic forms [4]. Additionally, the innovative value brought about by the digital economy is bound to exhibit marginal increments, exerting a potent permeation effect on the emphasis placed by regional economic resilience on innovation and transformative capacity, thereby providing an entirely new impetus. As a result, the driven economic resilience will feed into a more favorable environment for innovation, exerting a counterforce on the digital economy.

In summary, the two systems interact and exhibit interdependence in their development paths. The coordinated development of both the digital economy and regional economic resilience constitutes a crucial subject for global governance. However, current research regarding the digital economy and regional economic resilience predominantly focuses on the changes in individual system development and their respective driving forces. There needs to be more perspectives that elucidate the performance characteristics of coordinated development under the mutual influence of the digital economy and regional economic resilience. Additionally, a comprehensive evaluation system to assess the changing paths of the coordination relationship between the two systems remains to be clarified. Methods for calculating the performance of interactions between the two systems are still being explored, posing challenges to this study. Nevertheless, given the geopolitical development disparities, identifying the phased characteristics and evolutionary process of the coordinated development between the digital economy and regional economic resilience and discovering practical principles conducive to the joint governance of both systems holds significant importance for maintaining economic order and stability in different economic entities.

In conclusion, as global economic challenges continue to escalate, the discourse surrounding the digital economy and economic resilience is gradually becoming a focal point of scholarly attention. Seizing the new opportunities in digital economic development, contemplating the coordinated development of economic resilience, and the healthy, high-quality development of the digital economy constitute a crucial aspect of economic advancement. However, there is a need for further refinement in understanding how to study the inclusive and mutually beneficial system direction between the two, subsequently enhancing methods for improving economic order. To address this, this paper focuses on the collaborative development shifts between China’s digital economy and economic resilience, observes the coupling performance and path evolution mechanisms of the two systems under geopolitical disparities, and provides guidance for the enhancement and upgrading of regional economic systems. It also holds reference value for innovative economic forms and enhanced competitiveness in various countries.

This paper’s main contributions lie in several key aspects. Firstly, the current research on the relationship between digital economic development and regional economic resilience predominantly focuses on the unilateral impact of digital economic development on regional economic resilience. There is limited study on the synergistic effects and development characteristics of both. This paper innovatively shifts the focus to the collaborative development paths of the two systems and identifies their distinctive features. Secondly, a comprehensive evaluation index system for both the digital economy and regional economic resilience is constructed to reflect the developmental capabilities of both systems. Thirdly, guided by coupling theory, we employ a coupling coordination model to measure the interaction between the two systems, categorizing them based on different coupling coordination levels. This, combined with temporal-spatial distribution discussions, provides a reference for selecting research methodologies. Lastly, using Chinese regions as samples, we conduct spatial autocorrelation analysis and Geodetector to explore the characteristics of coordinated development from multiple perspectives. This enhances the research’s purpose and provides a basis for the efficient collaborative development of the digital economy and economic resilience in Chinese regions. Furthermore, it is an enlightening reference for research on similar themes in other regions worldwide.

## 2 Literature review

Tapscott (1996) is credited with introducing the concept of the digital economy, emphasizing the description of the digital technology industry and the productivity of the digital industry [5]. As research progressed, the digital economy has become a new economic form, succeeding the agricultural and industrial economies. The widely accepted definition in economics acknowledges the digital economy as a diverse and open economic structure centered around data as its fundamental element. The digital economy primarily depends on modern information networks as its main conduit and digital technological innovation as its driving force. This is achieved through the implementation of various new models and technologies within the realm of digitization [6]. Many academics have researched the digital transformation of the global economy and the quest for high-quality economic development within the context of the rapidly expanding digital economy worldwide [7–9]. These studies explore the impact of the digital economy on established economic sectors and investigate its potential to foster high-quality businesses. Both the "Fourteenth Five-Year Plan" and Vision 2035 of China prioritize fostering the overall growth of the digital economy. The objectives include accelerating the balanced expansion of the digital economy, facilitating its deep integration with the real economy, and nurturing it to become a strategic pillar for China’s high-quality economic development. The study of the digital economy goes beyond conventional logic, standards, and qualitative and quantitative research techniques in contrast to the traditional economy [10]. In order to address the shortcomings of conventional research methods in economic studies, it uses tools including extensive data analysis, digital modeling, and high-dimensional data [11]. The advent of numerous high-dimensional means and tools has also aided the improvement and advancement of conventional econometric techniques. These new tools make it possible to concentrate on data analysis and make it simpler to build accurate prediction models with great adaptable covariates [12]. The significant influence of the digital economy system’s performance has prompted scholars to investigate the radiation relationship between the digital economy and the traditional economic theory system. Researchers are examining the impact of the digital economy on the real economy, which encompasses the study of the "dandelion effect" [13]. Furthermore, researchers are examining ways to enhance regional economic resilience, increase resilience, and enhance the quality of development paths. Considering whether the economic environment can stimulate new economic growth momentum [14]. The growth of the digital economy has also produced a "long tail effect" and other consequential impacts on the development of local economies [15]. These issues are crucial for studying the theoretical framework of the digital economy and contribute to constructing a structural framework. Nevertheless, despite scholars’ extensive research, there has been relatively limited analysis from the perspective of the interaction between the digital economy and regional economic resilience. The research structure in this area needs further expansion, and the relevant system requires improvement to address these gaps.

The concept of regional economic resilience finds its roots in the study of ecological resilience, engineering resilience, and evolutionary resilience [16]. In the field of regional economics, early scholars, including Reggiani (2002), introduced this concept [17]. The early conceptualizations were derived from the emulation of natural ecosystems, applying the notion of "resilience" to illustrate the system’s ability to undergo self-repair and bounce back from external shocks [18]. Scholars have made further advancements in their research on regional economic resilience as the understanding of the concept has deepened. In particular, the ideas of disequilibrium and evolutionary theory in Evolutionary Economic Geography (EEG) have significantly shaped this research [19]. The theoretical framework and empirical studies on regional economic resilience have resulted in various viewpoints. Martin et al. (2016), known for their work on adaptability theory, propose that regional economic resilience lies in its ability to interact with complex systems such as the market, competition, and the environment [20]. Enabled by this resilience, the regional economic system can recover from shocks and readjust its development path, ultimately generating a new trajectory better adapted to its growth. Through research generalization, Martin et al. (2016) emphasize the continuity of regional economic resilience from four dimensions. Drawing on the adaptive cycle model, Simmie et al. (2010) categorize the evolutionary perspective of the regional economy into four stages: restructuring, development, maintenance, and release [21]. Emphasizing the dynamic nature of the regional economy, this framework highlights its capacity to adapt and transform as time progresses. Boschma et al. (2013) and other scholars have examined enhancing regional economic resilience by exploring new paths [22]. Utilizing spatial measurements, they analyze regional economic resilience from various perspectives, focusing on the intrinsic mechanisms that drive the update and development of regional industries within the context of the new trajectory. The recovery of economic production within China’s regions has become a crucial issue in the post-epidemic era. Consequently, there has been a significant surge in the study of regional economic resilience in China. Chinese scholars have proposed research frameworks and summaries based on their exploration of international studies on regional economic resilience [23,24]. Additionally, they have conducted empirical analyses and engaged in discussions on regional economic resilience from various perspectives. In their study, Song et al. (2022) analyzed China’s economic resilience characteristics, traced the evolution of this resilience, and explored the influencing factors behind its effects [25]. Researchers have also concentrated on understanding regional economic resilience mechanisms, a term with different spatial and temporal characteristics. The "region-economy-system" structure has been studied by several academics [26,27]. Evolutionary economic geography has extensively investigated the spatial spillover impact of regional economic resilience and the variables that influence it [28]. The recent development of research on regional economic resilience in China has led to differing views concerning establishing an indicator system. Most studies still rely on conclusions drawn from developed countries, often overlooking the heterogeneity among different regions within China, which is influenced by the external environment.

In conclusion, the current research on developing the digital economy and regional economic resilience has yielded some preliminary conclusions. However, there is still a need to strengthen the investigation of the cross-directional relationship between these two themes. The existing literature predominantly focuses on the one-sided impact of the digital economy on regional economic resilience [29,30], with limited research investigating how digital economic development influences changes in economic resilience and the coordinated role of these two factors. In the post-epidemic era, delving into the synergistic differences in regional economic resilience amid rapid digital economy development and assessing the alignment between economic resilience and the digital economic system in various regions can offer valuable guidance for countries’ economic recovery endeavors.

Therefore, building upon existing research, this study integrates the digital economy and economic resilience systems. It selects the 31 provinces in China (excluding Hong Kong, Macao, and Taiwan) as research samples for 2011–2020. The study analyzes the coupling relationships by constructing a comprehensive evaluation index system for both systems. Additionally, it employs spatial autocorrelation models and Geodetector to address the following questions: (1) What is the comprehensive development trend of both digital economic development and regional economic resilience in China? (2) How does the two systems’ temporal variation in coupling coordination manifest? What distinctive characteristics exist among different regions? (3) Does China’s level of coupled development exhibit spatial correlations, and what are its evolutionary features? (4) What significant differences exist in the impact of different driving factors on the changes in coupling coordination between the two systems? Investigating these questions will draw relevant conclusions, and corresponding discussion suggestions will be put forward.

## 3 Research methods

### 3.1 Entropy weight-TOPSIS method

Research on indicator empowerment methods for evaluating the digital economy and economic resilience can be categorized into subjective and objective approaches. Subjective empowerment commonly employs the Analytic Hierarchy Process (AHP) method. However, the final results of subjective empowerment may need more practical considerations and tend to be subjective. This paper employs the entropy value method for objective indicator empowerment to ensure accuracy and scientific rigor in the evaluation results and reduce the influence of solid subjectivity in the empowerment process. This approach minimizes interference from human factors and enhances the reliability of the empowerment process.

After assigning weights to each indicator of the digital economic development and regional economic resilience systems, the TOPSIS calculates the comprehensive development index for both systems. This method involves computing the positive and negative ideal solutions for the evaluation index system. A relative fitness degree is determined by comparing the relative distances between alternative solutions and the positive and negative ideal solutions. This fitness degree is then used to rank and evaluate the excellence of the comprehensive development index [31,32]. The steps for calculating the comprehensive development index for both systems are as follows:

1. Standardize the data of each indicator of digital economic development and regional economic resilience system.
2. The information entropy and weight of each indicator are calculated using the entropy method.

$$E_{j}=-\frac{1}{\ln n}\sum_{i=1}^{n}\frac{x_{ij}}{\sum_{i=1}^{n}x_{ij}}\ln\frac{x_{ij}}{\sum_{i=1}^{n}x_{ij}}$$ (1)

Where *E*_*j*_ denotes the entropy value of the *jth* indicator, *n* is the number of evaluation objects, and *X*_*ij*_ denotes the value of the *jth* indicator of the *ith* object after standardization.


$$W_{j}=\frac{1-E_{j}}{\sum_{j=1}^{m}\left(1-E_{j}\right)}$$ (2)

*W*_*j*_ denotes the weight of the *jth* indicator, and *m* is the number of indicators.

(3) Build a weighted matrix and calculate the positive and negative deviation square method as well as the positive and negative ideal solutions using the Euclidean distance [33].


$$Z={\left(z_{ij}\right)}_{n\times m}={\left(x_{ij}W_{j}\right)}_{n\times m}$$ (3)

$$z^{+}=\{\max(z_{1j},z_{2j},\cdots ,z_{nj})|j=1,2,\cdots ,m\}$$ (4)

$$z^{-}=\{\min(z_{1j},z_{2j},\cdots ,z_{nj})|j=1,2,\cdots ,m\}$$ (5)

In this context, *Z*_*ij*_ represents the weighted matrix value for the *jth* indicator of the *ith* object, while *Z*^+^ and *Z*^−^ denote the positive and negative ideal solutions for the *jth* indicator, respectively.

(4) Calculate the distance and proximity of each evaluation object to the positive and negative ideal solutions [34].


$$D_{i}^{+}=\sqrt{\sum_{j}{\left(z_{ij}-z^{+}\right)}^{2}}$$ (6)

$$D_{i}^{-}=\sqrt{\sum_{j}{\left(z_{ij}-z^{-}\right)}^{2}}$$ (7)

$$R_{i}=\frac{D_{i}^{-}}{D_{i}^{+}+D_{i}^{-}}$$ (8)

$D_{i}^{+}$ and $D_{i}^{-}$ represent the distance of the *ith* evaluation object to the positive and negative ideal solutions, respectively. *R*_*i*_ signifies the relative proximity of the *ith* solution to the ideal solution. These values are used as the corresponding values for both systems’ comprehensive evaluation development index.

### 3.2 Coupling coordination model

The concept of coupling originates from physics and is employed to quantify the interactions and influences between two or more systems. Introducing the coupling coordination model makes it possible to reflect the degree of coupling coordination between digital economic development and regional economic resilience. This model enables the assessment of the collaborative development situation between the two systems. The formula is as follows [35]:

$$C={\left[\frac{X\times Y}{{\left[(X+Y)/2\right]}^{2}}\right]}^{\frac{1}{2}}$$ (9)

T=αX+βY (10)

$$D=\sqrt{C\times T}$$ (11)

The degree of coupling is denoted by the variable C, whereas the comprehensive evaluation of digital economic development and regional economic resilience is represented by *X* and *Y*, respectively. *T* denotes the comprehensive coordination index between digital economic development and regional economic resilience systems. In this context, the weights assigned to the digital economic development and regional economic resilience systems are typically considered equally important. Therefore, the weight value is set as α = *β* = 0.5. The variable *D* represents the degree of coupling coordination, which reflects the coupling level between the two systems. Scholars have classified the coupling coordination degree [36–38] into eight categories, further divided into three levels, as illustrated in Table 1.

**Table 1:** Criteria for classifying the degree of coupling coordination.

Form	Type of coupled coordination	D-value
**Coordinated development**	High-level coordination	0.8–1.0
	Intermediate coordination	0.7–0.79
	Primary coordination	0.6–0.69
**Transitional development**	Barely coordinate	0.5–0.59
	Edge disorder	0.4–0.49
**Disorder development**	mild disorder	0.3–0.39
	moderate disorder	0.2–0.29
	severe disorder	0.0–0.19

### 3.3 Spatial autocorrelation model

Spatial autocorrelation examines regional variations and correlations by measuring spatial location, aggregation, and heterogeneity. The spatial autocorrelation model encompasses global spatial autocorrelation and local spatial autocorrelation.

#### 3.3.1 Global spatial autocorrelation

The global Moran’s I statistic is employed for measurement to analyze the overall spatial distribution of a specific attribute in the research area. The expression is as follows [39,40]:

$$I=\frac{n\sum_{i=1}^{n}\sum_{j=1}^{n}w_{ij}(x_{i}-\bar{X})(x_{j}-\bar{X})}{\sum_{i=1}^{n}\sum_{j=1}^{n}w_{ij}\sum_{j=1}^{n}{\left(x_{i}-\bar{X}\right)}^{2}}$$ (12)

In this context, *Ⅰ* represents the global Moran’s index, where *x*_*i*_ and *x*_*j*_ denote the coupling coordination degree between digital economic development and regional economic resilience in regions *i* and *j*, respectively. $\bar{X}$ represents the mean value of coupling coordination for all research areas. Additionally, n indicates the total number of study regions, and *w*_*ij*_ represents the spatial weight matrix.

#### 3.3.2 Local spatial autocorrelation

The local Moran’s I is employed to measure the coupling coordination degree value in each local space within the study area, considering the spatial correlation between this region and neighboring regions [39,40]:

$$I=\frac{x_{i}-\bar{X}}{\sqrt{\left(x_{i}-\bar{X}\right)}}\sum_{j=1}^{n}\left(x_{i}-\bar{X}\right)$$ (13)

The meaning of each index in the formula is the same as the global Moran’s I index.

### 3.4 Geo-detectors

Geodetector is a statistical method of driver detection analysis of drivers based on spatial dissimilarity, yielding a q-value reflecting the consistency of the spatial distribution pattern of the independent and dependent variables with the following formula:

$$q=1-\frac{\sum_{h=1}^{i}N_{h}\sigma_{h}^{2}}{N\sigma^{2}}=1-\frac{SSW}{SST}$$ (14)

Where *h = 1*,…,*i* denotes the stratification of variable *Y* or factor *X*, *N*_*h*_ and *N* are the number of cells in stratum *h* and the whole, respectively, $\sigma_{h}^{2}$ and σ^2^ are the variance of stratum *h* and the overall *Y* value. *SSW* and *SST* are the intra-layer variance and the overall total variance. *q* is taken as [0,1]; the closer *q* is to 1, the stronger the influence of factor *Y* and variable *X*, and the weaker the opposite is.

## 4 Evaluation indicators and data sources

### 4.1 Evaluation indicator system

Scholars have yet to reach a consensus on the evaluation index system for the digital economy, and an internationalized evaluation system for the digital economy has yet to be established. However, research on the evaluation system for the digital economy should prioritize practicality. Therefore, this paper, after considering the embedded relationship between the digital economy and various aspects of the economy, society, and daily life, as well as the current state of innovation and development in the digital economy, incorporates findings from the research on the evaluation system of digital economy development by Zhao et al. (2020) [41], Shen et al. (2022) [42], and Bai et al. (2021) [43]. It selects evaluation criteria from three dimensions: innovation-driven, level of development, and industry enhancement, encompassing nine influential elements. Regarding the current economic resilience evaluation index system, research can be broadly categorized into two approaches. The first approach, proposed by Martin et al. (2019), introduces a regional economic resilience evaluation system [44–46]. This system calculates the regional economic resilience index by selecting a single indicator that demonstrates sensitivity to economic resilience, such as GDP, GDP growth rate, data on the unemployed population, etc. This method is relatively mature and has been widely adopted by many scholars. The second approach takes a comprehensive perspective by considering various representative factors influencing economic resilience in the region. This involves establishing a complete indicator evaluation system and researching regional economic resilience using a combination of multiple indicators. This paper argues that while Martin et al.’s proposed evaluation system for the economic resilience of regional economies is highly practical and has been widely utilized in empirical research, a single indicator cannot fully capture the complexity of China’s economic resilience. Moreover, a significant disparity in economic openness exists among different regions, making reliance on a single indicator overly simplistic. Therefore, considering China’s economic development characteristics, we opt for multiple indicators to establish a comprehensive indicator system for evaluating economic resilience within regions from various dimensions. We refer to the research on regional economic resilience systems by Li et al. (2022) [47], Zhao et al. (2022) [48], and Zhu et al. (2020) [49] to establish a comprehensive evaluation system comprising fifteen elements under three quasi-measurement layers: defense and recovery Adaptation and conditioning, and innovation and transformation.

Building upon the insights above, a comprehensive indicator evaluation system is formulated to assess the interaction mechanism between economic resilience and digital economy development systems. The rationale for selecting specific indicators is elucidated based on the referenced literature, and detailed meanings and explanations are presented in Table 2.

**Table 2:** Comprehensive evaluation index system of digital economy development and regional economic resilience.

Evaluation systems	Levels of evaluation	Elements of evaluation	Evaluation indicators	Interpretation	Symbolic
Development of the digital economy	Innovation drive	Digital infrastructure	Number of Internet broadband access subscribers	This indicator represents the digital user base, reflecting the digital infrastructure [41,43]	Positive
		Digital environment	All-society fixed-asset investment in the information transmission, computer services and software industry	This indicator represents investment in fixed assets in digitization-intensive industries, reflecting the digital innovation environment [42,43]	Positive
		Elements of digital innovation	Expenditures on science and technology	This indicator indicates the capacity of science and technology input factors, reflecting the innovation factor gap [42]	Positive
	Level of development	Level of digital outputs	Number of digital economy-related patents per 10,000 people	This indicator represents the performance of digital innovation outputs and captures the level of digital outputs [42,43]	Positive
		Digital technology penetration	Websites per 100 businesses	This indicator indicates the distribution of digital technology development, reflecting the degree of penetration of digital technology development [43]	Positive
		Number of relevant employees	Employed persons in information transmission, software and information technology services/employed persons in urban units	This indicator represents the development of human capital in the digital economy, reflecting the percentage of the relevant workforce [41–43]	Positive
	Industrial enhancement	Digital Inclusive finance index	Peking University Digital Inclusive Finance Index	This indicator is a comprehensive representation of multiple dimensions under the digital finance umbrella and is widely used as an indicator for digital economy assessment [41–43]	Positive
		Industrial digitization	Number of computers per 100 persons in industrial enterprises	This indicator responds to the quality of digitization within the industry and reflects the degree of digitization enhancement in the industry [42,43]	Positive
		Industry digital outputs	E-commerce turnover of industrial enterprises and its share in the main business income of industrial enterprises	This indicator responds to the ability of industrial enterprises to apply digital output, reflecting the degree of improvement in the digital output of the industry [42]	Positive
Regional economic resilience	Defense and recovery	Economic development	GDP per capita	This indicator is representative of regional economic development and directly reflects changes in economic development [47,49]	Positive
		Social element	Urban unemployment rate	This indicator responds to the presence of the social labor force, reflecting the elements of the social labor force [49]	Negative
		Population structure	Population structure Percentage of population aged 15–64	This indicator expresses the socio-demographic quality, reflecting the demographic advantages and disadvantages [48,49]	Positive
		Industrial structure	Industrial structure rationalization index (Thiel index)	This indicator indicates the degree of rational distribution of industries, reflecting the framework of industrial structure [47–49]	Positive
		Income level	Per capita disposable income	This indicator expresses income disposability and captures disparities in income levels [49]	Positive
	Adaptation and conditioning	Financial structure	Year-end deposits of financial institutions/year-end loans of financial institutions	This indicator represents the ratio of deposits to loans of financial institutions and captures the resilience of the financial structure [49]	Positive
		Government regulation	Local fiscal expenditures	This indicator represents the financial strength of the government, reflecting the degree of government regulation [48,49]	Positive
		Sustainability level	Total investment in industrial pollution control as a share of fiscal expenditure	This indicator represents the share of governance inputs and captures the extent to which sustainable levels of development are regulated [48,49]	Negative
		Spending power	Consumption rate of the population	This indicator expresses the population’s willingness to consume, reflecting the gap in consumption capacity [48,49]	Positive
		External link	Foreign direct investment as a share of GDP	This indicator represents external investment, reflecting gaps in access to external financial support [47–49]	Positive
	Innovation and transformation	Technological progress	R&D investment	This indicator shows the investment in scientific research, reflecting the basic capacity for technological progress and innovation [49].	Positive
		Educational level	Percentage of college degree and above	This indicator expresses the quality of the level of education, reflecting the gap in the level of education [48,49]	Positive
		Industrial upgrade	Percentage of tertiary sector value added	This indicator indicates the proportion of tertiary industry upgrading, reflecting the ability of industrial transformation and upgrading [47–49]	Positive
		Environmental governance	Emissions per unit of Sulphur dioxide	This indicator captures emissions of environmental pollutants and reflects the effectiveness of environmental governance transformation [48,49]	Negative
		Innovations	Number of patents granted to 10,000 people	This indicator reflects the number of patents reached for authorization and the ability to implement the results of innovation [49].	Positive

### 4.2 Data processing

The raw indicators were standardized to eliminate scale differences, considering variations in the impact of different hands on the two systems. These indicators were then categorized into positive and negative groups based on their respective contributions to the system. The following processing method was employed:

$$\mu_{ij}=\frac{x_{ij}-\min[x_{j}]}{\max[x_{j}]-\min[x_{j}]},\;\mathrm{Positive};$$ (15)

$$\mu_{ij}=\frac{\max[x_{j}]-x_{ij}}{\max[x_{j}]-\min[x_{j}]},\;\mathrm{Negative};$$ (16)

Where *μ*_*ij*_ is the normalized value; *x*_*ij*_ is the initial value of the *jth* indicator in year *i*; max *x*_*j*_ is the maximum value of the *jth* indicator; min *x*_*j*_ is the minimum value of the *jth* indicator.

The industrial structure rationalization index is represented by the modified Thiel index, which is an index originally proposed by Theil to measure regional income disparity or inequality. This index is widely used in studies [50] to assess the level of rationalization in the industrial structure. The formula for calculating the modified Thiel index is as follows:

$$TL=\sum_{i=1}^{n}\frac{Y_{i}}{Y}\ln[(\frac{Y_{i}}{L_{i}})/(\frac{Y}{L})]$$ (17)

Where *Y*_*i*_ represents the total output value of the *ith* industry, *L*_*i*_ denotes the total employment of the *ith* industry, *Y* denotes the total output value, and *L* means the total employment. When *TL* equals zero, the economy is in equilibrium, and the structure is considered more irrational as the *TL* value increases.

### 4.3 Data sources

Given the concepts of digital economy and regional economic resilience have emerged relatively recently, it is crucial to acknowledge that early statistics and data might need more completeness. This study focuses on the 31 provinces, autonomous regions, and municipalities directly under the central government of China. The selected data for analysis encompasses the years 2011 to 2020 and includes pertinent indicators of digital economic development and regional economic resiliency.

The data used in this study are derived from several official publications, which include the China Statistical Yearbook, China Urban Statistical Yearbook, China Electronic Information Industry Statistical Yearbook, China Science and Technology Statistical Yearbook, and provincial statistical yearbooks from 2011 to 2020. Additionally, the digital financial inclusion index data are obtained from the Digital Finance Research Center of Peking University and the open research platform on digital finance provided by the Ant Financial Services Group. The interpolation method is employed to address any missing data, allowing for the estimation of values for the missing data points.

## 5 Comprehensive development level analysis

**Fig 1:**
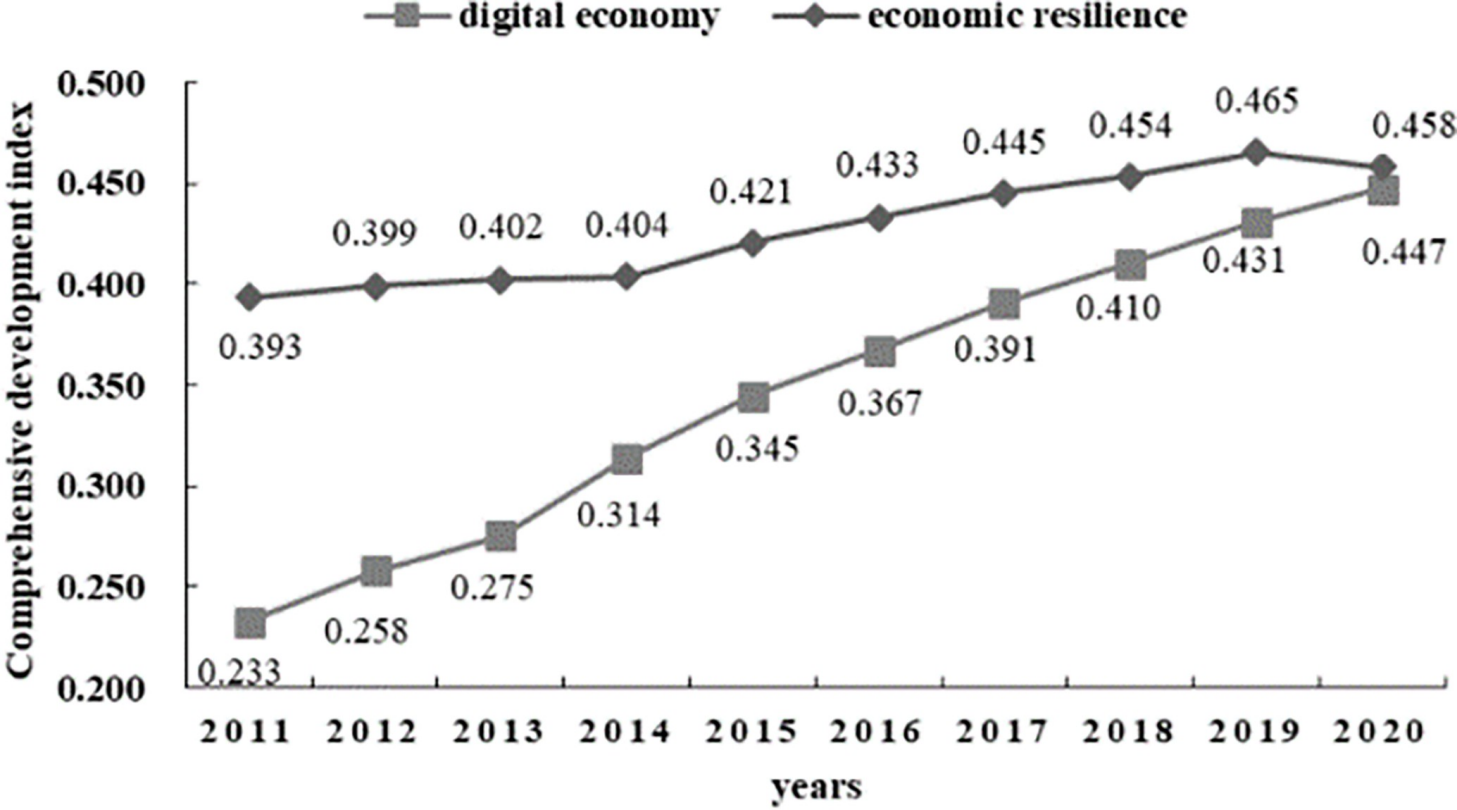
Comprehensive development index trend.

In this study, the data processing of multiple indicators related to digital economic development and regional economic resilience in China’s comprehensive development relies on the entropy weight-TOPSIS method. This analysis enables us to examine the composite development index’s trend evolution between 2011 and 2020. Fig 1 depicts the results.

From the data illustrated in Fig 1, it is clear that China’s digital economy witnessed substantial growth in its overall level of advancement from 2011 to 2020. The general development index has exhibited significant growth, increasing from 0.2329 in 2011 to 0.4580 in 2020 at a rate of 49.15%. The development index has consistently demonstrated sustained positive growth throughout the research period, indicating a promising trend in China’s digital economy’s development process. This positive trend highlights the strong driving force of innovation, continuous advancements in the degree of development, and a significant increase in the digital industry’s production capacity. Concerning regional economic resilience, the 2011–2020 progress trend curve exhibits a steady ascent. In contrast to the "leapfrog" increase in the digital economic development index, regional economic resiliency has grown 12.1% more slowly. Regional economic resilience reflects how the regional economy evolves under various external and internal factors. The continuous increase in economic resilience indicates the growth of the economic system. As the second-largest economy in the world, China maintains a substantial economic volume and reliable economic support, which creates favorable conditions for further economic growth. It is worth noting that the Economic Resilience Index experienced a slight decline in 2020. Considering the backdrop of the COVID-19 outbreak in China during this period, the economic system demonstrated resilience by making continuous adjustments to ensure a successful recovery from the crisis and maintain self-stability [51]. However, various elements that provide resilient operation were somewhat hindered by the crisis’s impact, thus limiting the development of economic resilience and causing a downturn.

## 6 Coupling coordination performance analysis

### 6.1 Time-series characteristics

A coupled coordination model is utilized to calculate the comprehensive development index of digital economic development and regional economic resilience, enabling the analysis of interaction between the two research targets. This calculation aims to identify the coupled and coordinated time-series characteristics of China’s 31 provinces and municipalities concerning digital economic development and regional economic resilience between 2011 and 2020.

**Fig 2:**
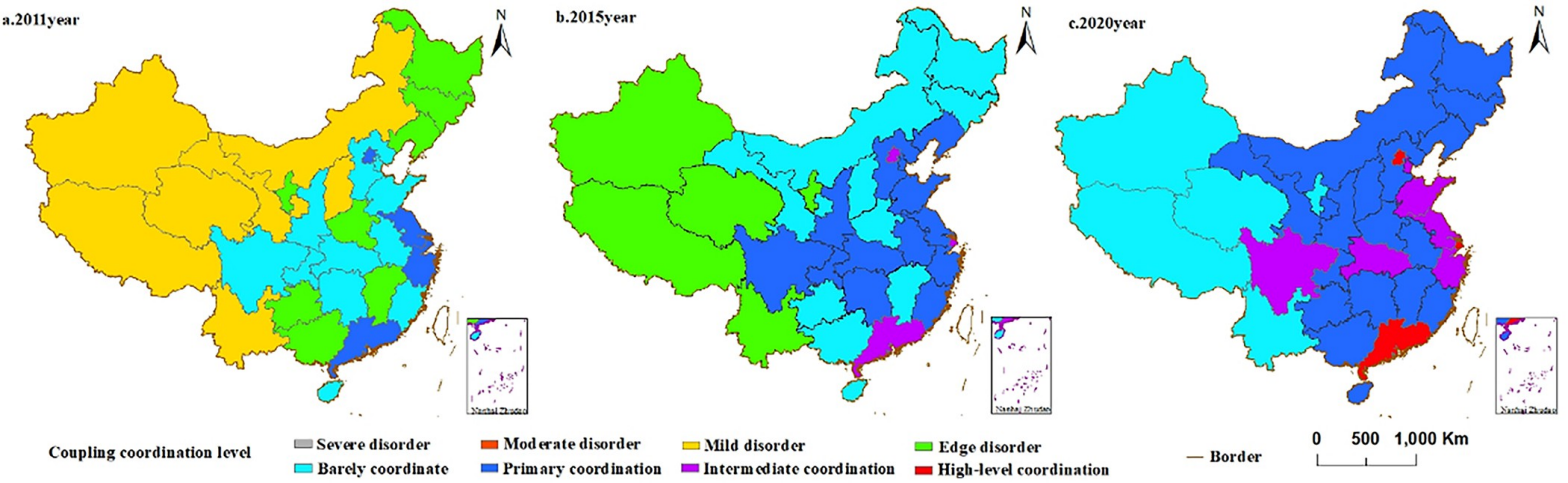
Coupled coordinated horizontal distribution evolution (the administrative boundaries of Provincial-level are republished from [52] under a CC BY license, with permission from the resource and environment data centre, original copyright 2022).

Throughout the research period, the coupling degree between China’s digital economic development and regional economic resilience has consistently remained above 0.8, as determined using Formula (9). When the coupling degree approaches 1, it indicates a convergence to an ordered state, demonstrating a positive and significant interaction between the two systems. Formula (11) is also applied to determine the degree of coupling coordination between the two systems. The changes in the national coupling coordination degree across the study period are matched to the corresponding intervals based on the predefined division standards established in this study. Due to space limitations, the 2011, 2015, and 2020 calculations are selected for visualization using ArcGIS10.2 software, as depicted in Fig 2.

The coupled coordination degree between China’s digital economy development and regional economic resilience has consistently improved throughout the study period. The least favorable performance observed during this entire process was only mild disorder, without any instances of moderate disorder or severe disorder. Upon calculating the average annual growth rate (refer to Table 3), it is evident that the degree rose from 0.504 in 2011 to 0.658 in 2020, signifying an average yearly growth rate of 3.01%. This suggests a notable mutually reinforcing effect within the development system of China’s digital economy and regional economic resilience. Upon closer examination of the results of coupling coordination degree calculations, it is worth noting that Xinjiang and Tibet demonstrated the lowest values during the research period, registering at 0.455. These values placed them on the edge of the disorder level. Upon further investigation of the sample size for this lowest value, it was found that in the province of Tibet in 2011, the performance was 0.313, indicating a state of mild disorder. However, the growth rate was 11.14%, signifying an ongoing improvement in the coordination effect between the digital economy and regional economic resilience. An observation of the growth rates in coupling coordination degree for other provinces and municipalities reveals that the remaining performances were positive apart from isolated negative values observed towards the end of the study period. This indicates that during the study period, the coupling coordination of development between the digital economy and regional economic resilience shifted from disorder development to transitional development and Coordinated development, displaying an upward trajectory. It underscores the mutually beneficial dynamic between developing China’s digital economy and regional economic resilience.

**Table 3:** Comprehensive index proportion and coupling coordination degree growth rate changes.

Regions	Growth rate			Composite index ratio		
	2011–2012	2015–2016	2019–2020	2011	2015	2020
**Beijing**	3.03%	1.49%	-0.42%	0.714	0.916	1.088
**Tianjin**	3.43%	2.59%	0.43%	0.551	0.787	0.898
**Hebei**	2.21%	3.45%	0.59%	0.516	0.797	0.967
**Shanxi**	6.26%	10.30%	0.58%	0.547	0.769	0.919
**Inner Mongolia**	4.61%	8.58%	0.99%	0.590	0.910	0.994
**Liaoning**	6.31%	2.65%	-0.07%	0.433	0.802	1.015
**Jilin**	8.11%	1.61%	1.09%	0.530	0.776	0.958
**Heilongjiang**	1.43%	2.02%	0.80%	0.395	0.786	0.981
**Shanghai**	3.26%	0.97%	5.06%	0.709	0.907	1.000
**Jiangsu**	3.25%	2.04%	1.30%	0.657	0.813	0.962
**Zhejiang**	3.28%	1.66%	0.75%	0.678	0.840	1.014
**Anhui**	1.98%	2.02%	0.65%	0.676	0.850	1.008
**Fujian**	3.26%	2.60%	0.13%	0.645	0.820	0.929
**Jiangxi**	2.88%	6.87%	1.06%	0.643	0.813	0.923
**Shandong**	3.27%	1.97%	0.89%	0.524	0.823	0.978
**Henan**	5.54%	3.19%	1.65%	0.490	0.683	0.940
**Hubei**	2.03%	2.14%	0.92%	0.546	0.793	1.093
**Hunan**	3.51%	2.58%	1.47%	0.572	0.820	0.898
**Guangdong**	3.03%	2.48%	1.26%	0.619	0.797	1.025
**Guangxi**	6.79%	8.58%	1.58%	0.537	0.575	0.842
**Hainan**	3.18%	6.70%	1.04%	0.803	0.991	0.967
**Chongqing**	3.22%	3.22%	-0.71%	0.502	0.729	0.947
**Sichuan**	2.72%	2.42%	1.91%	0.601	0.797	0.948
**Guizhou**	5.24%	5.45%	1.57%	0.572	0.743	0.958
**Yunnan**	8.21%	3.23%	3.94%	0.623	0.870	0.983
**Tibet**	11.14%	11.28%	-2.99%	0.433	0.926	1.103
**Shaanxi**	3.57%	2.27%	1.00%	0.676	0.836	1.035
**Gansu**	3.98%	12.56%	0.52%	0.577	0.818	1.010
**Qinghai**	7.49%	3.44%	0.68%	0.634	0.914	0.992
**Ningxia**	2.51%	2.53%	2.12%	0.778	0.979	1.012
**Xinjiang**	3.61%	2.74%	-0.02%	0.490	0.726	0.859
**Mean**	4.03%	3.82%	0.98%	0.589	0.820	0.976

Fig 2 also shows that the coupling and coordination levels between the digital economic and regional economic resilience vary annually across provinces. To expound upon this phenomenon, we further calculate the ratio of the development indices for the digital economy and regional economic resilience. When the ratio equals 1, it indicates synchronous development; if it is less than 1, it signifies that the digital economy lags in comparison to the resilience development of the regional economy. By scrutinizing the index weights (see Table 3), we discern that the mean proportions for each province over the survey period have risen from an initial value of 0.589 to a final value of 0.976. Additionally, the ratios for different regions and cities have all experienced an augmentation, ultimately approaching 1. This suggests a transformation from the scenario where the digital economy lags in developing economic resilience to a state of synchronized development. This indicates that under the backdrop of orderly development in regional economic resilience, an advantageous environment and support system have been provided to enhance the digital economy. Simultaneously, the digital economy, relying on the resilience of the regional economy, exerts a more robust impetus. With the rapid advancement of the digital economy, a virtuous cycle of synchronous effects with regional economic resilience is generated, leading to a continuous improvement in the coupling and coordination degree between the two systems in each region.

Analyzing the chronological changes, it should be emphasized that in 2011, the four areas of Beijing, Shanghai, Guangdong, and Jiangsu displayed the highest degree of coupling coordination, indicating a primary level of coordination between the digital economy and regional economic resilience in these regions. This shows that the two systems in these areas have developed an appropriate degree of coordination and interaction, with each system having a favorable influence on the other. In contrast, the western and northeastern regions have predominantly shown mild disorder and edge disorder. At the same time, the central part of the country has mainly achieved a barely coordinated status in terms of coupling coordination. In 2015, China experienced significant development in the distribution of the coupling coordination degree. While Beijing, Shanghai, and Guangzhou advanced to the intermediate coordination stage, continuing to remain a key factor influencing the overall development direction. In the western region, Xinjiang, Tibet, Qinghai, Yunnan, and Ningxia remained at the edge disorder stage. However, Inner Mongolia, Gansu, and Shanxi, which were initially in the same echelon, experienced rapid improvement in their degree of coupling coordination, reaching the stage of barely coordinated. The eastern coastal region, the Yangtze River Basin, and some central provinces achieved a primary coordination stage in their coupling coordination degree. This transition reflects a shift from transitional development to coordinated development. In 2020, the coordination degree of linkage between the two systems showed further advancement. By this point, a strong correlation between the digital economy’s growth and local economies’ resilience was demonstrated as more than two-thirds of China’s provinces had entered the stage of coordinated development. Beijing, Shanghai, and Guangzhou achieved high-level coordination in their development, while Shandong, Jiangsu, Zhejiang, Hubei, and Sichuan reached the intermediate-level coupling coordination development stage. Observing the inter-regional differences in coupling coordination degree, a clear "east high, west low" ladder-type distribution is evident. In 2011, the distribution showed characteristics of barely coordinated, edge disorder, and mild disorder from east to west. By 2015, the distribution shifted to primary coordination, barely coordinated, edge disorder, and mild disorder. In 2020, the distribution further evolved to intermediate coordination, primary coordination, and barely coordinated from east to west.

### 6.2 Characteristics of locational differences

China’s digital economic development and regional economic resilience exhibit a distinct distribution of coordination from east to west, displaying notable agglomeration characteristics. To further examine how the two systems’ spatial connectivity and coordination are distributed, the concept of the eight integrated economic zones, as proposed by Hong [53], is introduced. This division allows for regional analysis of the 31 provinces, enabling a comprehensive and concentrated investigation of how the coupling coordination degree varies within each agglomeration. Based on the characteristics of the coupling coordination degree, this method facilitates the development of regionally tailored policies.

**Fig 3:**
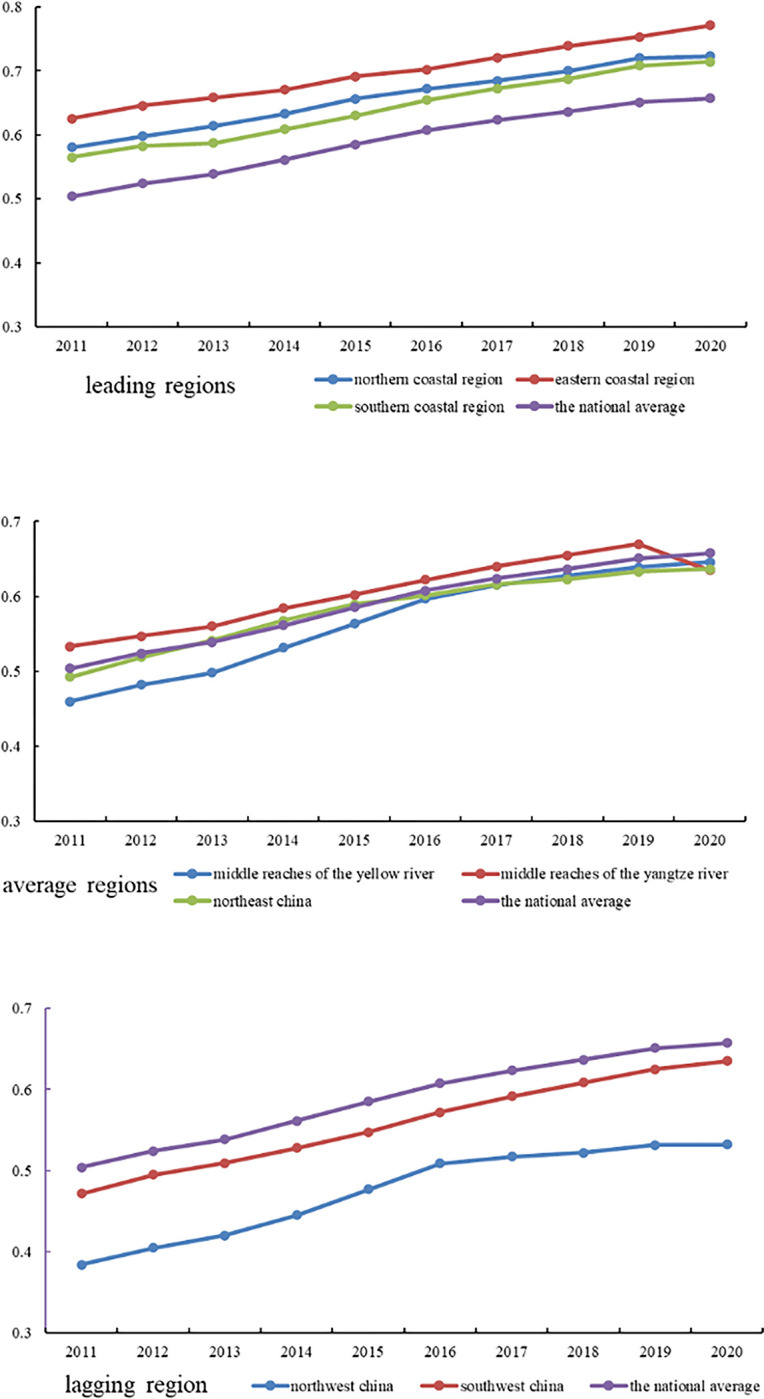
Development trend of coupling coordination degree at different levels.

Through a comparative analysis of the coupling and coordination trends in each comprehensive economic zone with the national average, we categorize the eight regions into three levels: leading, average, and lagging. It is worth noting that the general trend in all regions is steady improvement, with most falling within the [0.5–0.8] range. Further details regarding the varying trends among regions of different levels are illustrated in Fig 3.

The leading region encompasses the eastern coastal region, the northern coastal region, and the southern coastal region, with an average coordination coupling value of 0.666. Over the study period, the eastern coastal region exhibits the highest level of coupling coordination in the eight major economic complexes. Its coupling falls primarily within the range of primary and intermediate coupled development, representing a stage of coordinated development. Moreover, this region’s degree of coordination coupling significantly surpasses other comprehensive economic zones. Within the eastern coastal economic zone, Shanghai, Jiangsu, and Zhejiang demonstrate higher digital economy development indices, resulting in a narrower gap in regional economic resilience among them. This, in turn, fosters a robust interaction between the digital economy development system and the regional economic resilience system. Following closely in second and third place are the northern and southern coastal regions, characterized by development intervals ranging from barely coupled to primary coupled coordination. Taking a comprehensive view of the top three regions, it is evident that they are all coastal areas. Their development processes exhibit a relatively balanced similarity, with geographic location and openness offering distinct advantages for advancing the digital economy development system, resulting in significant progress. Simultaneously, the concentration of talent and industries further bolsters the innovation and transformative capabilities of the economic resilience system. These regions exemplify both systems’ harmonious coupling, interaction, and coordination, fostering mutually supportive development by reasonably allocating quality resources.

The second level encompasses the average regions, including the middle reaches of the Yangtze River, the Northeast region, and the middle Yellow River. The mean value of coupling coordination in these regions is 0.586. Compared to the national average level of coupling coordination, the middle reaches of the Yangtze River are above average. Within this region, the coupling coordination is at a state of barely coupled coordination to primary coupled coordination. The middle reaches of the Yangtze River region are influenced by the radiation effects of the eastern and southern coastal areas regarding its location. It receives the spillover of resources brought about by the agglomeration effect of the digital economy industry. And this region complements the digital economy’s development downstream of the Yangtze River. The digital industry’s orderly development and relatively stable resilience support regional economic resilience. It’s important to note that by the end of the research period, the coupling coordination level in the middle reaches of the Yangtze River showed a decline. This period coincided with the outbreak of COVID-19 in Wuhan, indicating that the virus’s impact led to a disruption in the coordination between the digital economy and regional economic resilience. The Northeast comprehensive economic region has some overlaps with the national average level—the coupling interval ranging from edge disorder to barely coupled to primary coupled coordination. As a substantial base for China’s secondary industry, the Northeast comprehensive economic region has experienced slow industrial structure adjustments and faces challenges in reform. This has led to the squeezing of the development of digital industries within the region, which is difficulty in obtaining quality investment support, thereby limiting their level of development. However, the region is characterized by large-scale state-owned enterprises, providing a certain level of support for regional economic resilience. Additionally, the innovation of internal digital systems within these enterprises has also propelled the development of the digital economy. The coupling coordination in the middle reaches of the Yellow River falls into edge disorder, barely coupled coordination, and primary coupled coordination. Although it did not stand out compared to other regions in terms of coupling development levels during the research period, it achieved the highest growth rate in coupling coordination at 28.8% among the eight comprehensive economic regions. In the initial stages of the research period, Inner Mongolia, Shanxi, and Shaanxi in the Yellow River middle reached the economic region needed to catch up to the national level regarding digital economic development. However, with the deepening of the "Central Rise" strategy and the introducing of the high-quality development strategy for the Yellow River basin, the economic resilience of the Yellow River middle reaches economic region has been effectively enhanced. This, in turn, has driven the development of the digital economy, leading to coordinated progress between the two systems and a rapid increase in coupling coordination.

The third level comprises the lagging regions, including the Southwest and Northwest regions, with a mean coupling coordination of 0.516. The coupling development range in the Southwest comprehensive economic region falls into edge disorder, barely coupled coordination, and primary coupled coordination. It achieved a coupling coordination growth rate of 25.7%, second only to the Yellow River middle reaches economic region. The rapid development of the Chengdu-Chongqing economic zone has played a driving role in the entire comprehensive economic region. Moreover, being conveniently located for cooperation between the upper and lower reaches of the Yangtze River and benefiting from the construction of the New Silk Road, the Southwest comprehensive economic region has gained new vitality. The development environment for the digital economy continues to improve, absorbing transformation ideas from the eastern regions. This has enhanced the region’s innovative and transformative capabilities of economic resilience. The level of coupling development between the two systems in the northwest region significantly lags behind other regions. The coupling coordination falls within the range of mild disorder to Barely coordinate, with the study period failing to break through to the coordinated development stage. Most areas within this integrated region experience uneven economic development, characterized by weak adaptive and regulatory capacity and limited acceptance of external resources. The regional economic resilience and the level of digital economy development in this region are below the national average. The scarcity of digital enterprises hampers the formation of an effective digital transformation, imposing significant constraints on the digital development level of Northwest China. As a result, it is crucial to carefully consider local conditions and prioritize the development of industries that leverage regional characteristics. By stimulating economic vitality, we can strengthen the region’s economic resilience and, in turn, promote the advancement of the digital economy through a dual-system mechanism. It is imperative to avoid adopting a catch-up strategy unquestioningly but rather tailor approaches to the specific circumstances of the region.

**Fig 4:**
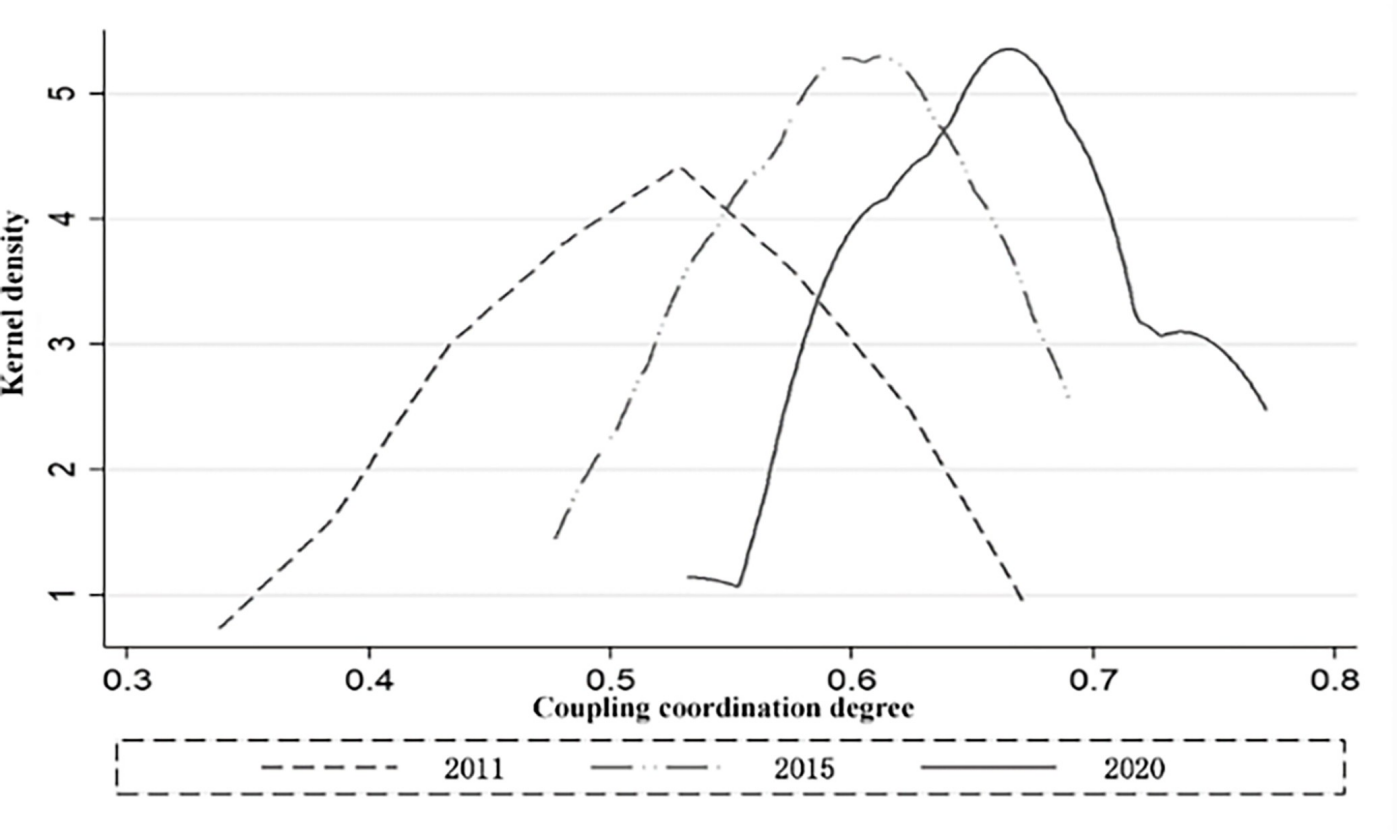
Distribution curve of kernel density in the eight integrated economic zones.

To further explore the overall distribution dynamics and evolutionary patterns of coupling coordination in the comprehensive economic regions, the kernel density estimation method is employed to create dynamic evolution trend maps of the coupling coordination between digital economic development and regional economic resilience in 2011, 2015, and 2020 (see Fig 4). The analysis examines the distribution trends, polarization tendencies, and distribution extensibility. Fig 4. shows that the coupling coordination degree’s kernel density curve’s center consistently shifts to the right. The data depicts a consistent upward trend in the central range of the coupling coordination level between regional economic resilience and digital economic development across China’s eight Comprehensive Economic Zones (CEZs). Moreover, the change interval of the kernel density curve decreases, suggesting a narrowing of the overall gap in the coupling coordination degree. The curve’s "tip" flattens, and its "peak" rises, showing a widening gap in the degree of coupling coordination between the eight comprehensive economic zones. As time progresses, the density curve in 2020 begins to display a trailing phenomenon, indicating a tendency to form a side peak. This shows that, among the eight comprehensive economic zones, there is a growing disparity in the degree of linkage coordination between the regions with a high level of digital economy development and those with a low level. Regions with a coupling coordination degree of 0.7 or higher tend to be found in Intermediate coordination, leading to a polarization phenomenon.

### 6.3 Characteristics of the spatial evolution

#### 6.3.1 Global spatial autocorrelation analysis

The degree of coupling coordination between digital economy development and regional economic resilience across the 31 provinces and municipalities was measured using the global Moran’s I index. During the study period, Table 4 shows that the global Moran’s I index for the coupling coordination degree is greater than 0 for all provinces and municipalities. Moreover, the Z value exceeds 1.96, and the P value is less than 0.01. These findings indicate the rejection of the original hypothesis "there is no spatial autocorrelation" at a significant level of 1%. The P-value of less than 0.01 further confirms the rejection of the initial hypothesis of "no spatial autocorrelation" at a 1% significant level. This suggests the presence of positive spatial correlation, explicitly indicating the spatial characteristics of "areas with high values of coupling coordination degree being surrounded by areas with high values" and "areas with low values of coupling coordination degree being surrounded by areas with low values." Upon observing the changes in Moran’s I index, it is apparent that from 2011–2020, the index is mostly higher than 0.3, except for 2016, when the value is lower at 0.285. This suggests that there are evident clustering characteristics in the coupling coordination of the growth of the digital economy and regional economic resilience. The clustering level starts low and then increases, displaying a "U" type distribution.

**Table 4:** Global Moran’s I Index.

Year	2011	2012	2013	2014	2015	2016	2017	2018	2019	2020
**Moran’s I**	0.355	0.347	0.355	0.348	0.310	0.285	0.314	0.338	0.348	0.349
**Z**	3.562	3.481	3.550	3.491	3.152	2.943	3.209	3.439	3.520	3.539
**P**	0.002	0.000	0.001	0.003	0.005	0.002	0.000	0.001	0.001	0.002

#### 6.3.2 Local spatial autocorrelation analysis

**Fig 5:**
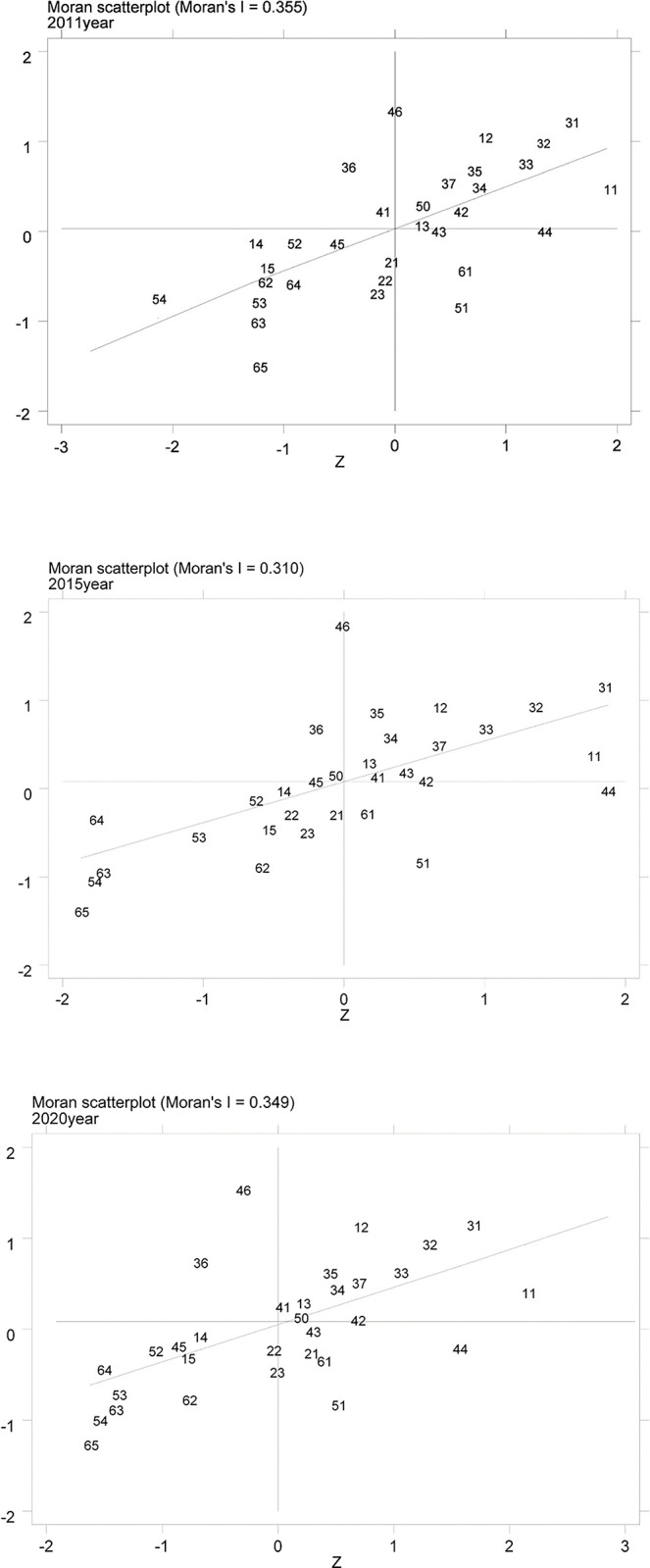
Local Moran’s I scatter distribution of the coupled coordination of digital economy development and regional economic resilience in 2011, 2015 and 2020, the Chinese provinces in Fig 5 are represented by the codes of the "Rules for the Compilation of Statistical Division Codes and Urban and Rural Division Codes (2022)" issued by the National Bureau of Statistics (NBS), which are as follows:11 Beijing, 12 Tianjin, 13 Hebei, 15 Inner Mongolia, 21 Liaoning, 22 Jilin, 23 Heilongjiang, 31 Shanghai, 32 Jiangsu, 33 Zhejiang, 34 Anhui, 35 Fujian, 36 Jiangxi, 37 Shandong, 41 Henan, 42 Hubei, 43 Hunan, 44 Guangdong, 45 Guangxi, 46 Hainan, 50 Chongqing, 51 Sichuan, 52 Guizhou, 53 Yunnan, and 54 Tibet,61 Shaanxi, 62 Gansu, 63 Qinghai, 64 Ningxia, 65 Xinjiang.

This work used the local Moran’s I index to analyze the spatial correlation variations among areas in the 31 Chinese provinces and municipalities. during the study period. Moran scatter plots were created using the 2011, 2015, and 2020 results, highlighting the coupling coordination characteristics of the different regions concerning themselves and their neighboring regions. The results of these plots are displayed in Fig 5.

From Fig 5, the first and third quadrants of the scatter plot, which represent the H-H (high-high) agglomeration and L-L (low-low) agglomeration, respectively, clearly show the main distribution of the digital economy development and regional economic resilience in the 31 provinces and municipalities of China. However, there is also a distribution in the second quadrant (L-H) and the fourth quadrant (H-L). These distribution characteristics indicate a significant positive local spatial correlation between regions, with bipolar agglomeration being the dominant pattern. In 2011, there were 13 regions in the third quadrant, but by 2020, the number of regions in the third quadrant decreased to 10. This suggests that the migration pattern is primarily characterized by upstream migration. The number of regions in the first quadrant (H-H) remains stable at 12, with the exception of Hainan, which experiences downstream migration. Hubei, on the other hand, reaches this level through upstream migration. This indicates a relatively stable spatial pattern among the regions. By analyzing the migration-prone areas, one can study the spatial evolution of the coupling coordination between the two systems in those regions.

1. Upstream migration: Liaoning, Jilin, and Heilongjiang provinces transitioned from L-L (low-low) agglomeration in the early part of the study period to H-L (high-low) agglomeration in the later part. The evidence indicates an enhancement in the coupling coordination level between the local digital economy’s growth and the economy’s resilience. The Northeast Economic Zone’s neighborly structure has allowed consistent spillover effects to benefit their development. Henan Province moved from L-H (low-high) agglomeration to H-H (high-high) agglomeration, indicating an improvement in its level of coupling and coordination. This improvement can be attributed to the radiation effect of leading regions and the absorption of opportunities for growth through spillover effects. Hunan Province transitioned from H-L (high-low) agglomeration to H-H (high-high) agglomeration in 2015. Still, towards the end of the research period, it returned to H-L (high-low) agglomeration. This suggests instability in the inter-regional development, indicating that its spillover effect could not sustainably drive the growth of neighboring regions effectively.
2. Downstream migration: Hainan Province transitioned from H-H (high-high) agglomeration at the beginning of the study period to L-H (low-high) agglomeration at the end. This indicates that Hainan Province did not maintain a consistent development trajectory between the digital economic development and regional economic resilience systems. Despite its geographic location in the southern coastal region and its adjacency to Guangdong Province, which provides a natural advantage for cooperation and opening up, Hainan Province needs to strengthen the utilization of resources further and improve its development situation to achieve significant breakthroughs.
3. Local migration: During the study period, most regions in the first quadrant, such as Beijing, Shanghai, Jiangsu, Tianjin, Zhejiang, Shandong, Fujian, Anhui, Hebei, and Chongqing, experienced local migration characterized by coupling development in leading regions or core driving areas. These regions demonstrated a higher level of coupling coordination, with stable performance in both systems. Jiangxi Province, situated in the second quadrant, encountered industrial barriers that impeded the growth of the digital economy, leading to a delayed pace of advancement and challenges in enhancing its efficiency. The provinces in the third quadrant are predominantly located in the northwest and southwest regions. These regions exhibit lower values of coupling coordination, indicating a "collapse zone" in inter-regional development. Acquiring high-quality resources and benefiting from the radiation effects of leading regions pose challenges, making it difficult for these regions to reach a higher level of coupling and coordination. However, the distribution of each region displays a tendency to the right when comparing the changes throughout the entire study period, showing an improvement in the coupling coordination level. In the fourth quadrant, Guangdong, Sichuan, and Shaanxi are development engines within their respective comprehensive economic zones, demonstrating good growth. However, the impact on other regional provinces and municipalities is limited, resulting in a high-low (H-L) agglomeration pattern. During the study period, Hubei Province changed its agglomeration pattern. It started in the high-high (H-H) category, shifted to high-low (H-L) in the middle, and finally reverted to high-high (H-H) agglomeration by the end. This indicates that Hubei Province has influenced the development of surrounding regions through its spillover effect. Situated in the middle sections of the Yangtze River, Hubei Province benefits from convenient transportation and abundant high-quality science and education resources, providing good development opportunities for neighboring areas.

### 6.4 Driver analysis

Various driving forces influence the coupling between the digital economy and economic resilience. In this study, geographic detectors are employed to analyze these factors. Based on existing research, the following variables are considered as detection factors: D1: GDP per capita (measured in 10,000 yuan), D2: length of fiber optic cables (measured in kilometers), D3: online payment capacity (measured in 10,000 yuan), D4: proportion of value added in the second and third industries (%), D5: population density (measured in people/km2), and D6: total amount of imports and exports of foreign-funded enterprises (measured in million dollars).To process the independent variables, the natural break method of ArcGIS software is utilized for type-volume transformation. The detection calculations for each driving factor yield the corresponding P-values. Due to space constraints, the detection results for 2011, 2015, and 2020 are listed in Table 5.

**Table 5:** Detection results.

Year	D1	D2	D3	D4	D5	D6
2011	0.4153**	0.6836**	0.5674***	0.3382*	0.2427*	0.6581***
2015	0.2960**	0.6811*	0.5995***	0.6357**	0.1600**	0.8434**
2020	0.3742***	0.7792***	0.6002***	0.5769*	0.2879**	0.8635**

Note

***, **, * indicate significant at 1%, 5%, and 10% level of significance, respectively.

The results in Table 5 show that each influencing factor demonstrates a significant relationship at different confidence levels across the three years. Furthermore, all the factors positively influence the coupling coordination degree between digital economic and regional economic resilience. Specifically, the online payment capacity stands out as a significant factor at a 1% confidence level throughout the study period, indicating its consistent and stabilizing impact on the coupling and coordination degree. Apart from online payment capacity, foreign-funded enterprises’ total amount of import/export also exhibited significant influence at a 1% confidence level in 2015. Population density and the value-added share of the second and third industries both exhibited growing influence in 2015, while the influence of total import/export of foreign-funded enterprises and fiber optic cable length decreased. In 2020, the significance of the influence of GDP per capita and fiber optic cable length increased, making them significant factors alongside online payment capacity. However, the extent of its effect is diminishing, as evidenced by the amount of value created in the second and third industries. These findings imply that several variables influence how closely the growth of the digital economy and regional economic resilience are coupled and coordinated, with online payment capacity consistently having a significant impact. The influence of other factors may vary across different years, reflecting the changing dynamics of the two systems.

The most influential factor, with P-values of 0.6581, 0.8434, and 0.8635 across the three years, is the total import and export amount of foreign-funded enterprises. The suggested strong relationship exists between the level of coupling coordination of the digital economy’s growth and regional economic resilience and foreign-funded firms’ total import and export quantity. It reflects the level of regional openness, as the growth of the digital economy often relies on the degree of openness, and regional economic resilience benefits from increased openness. Online payment capacity and fiber optic cable length consistently increase their P-values, reaching 0.7792 and 0.6002, respectively, in 2020, and are significant at a 1% confidence level. The growth of the digital economy is closely linked to these two elements. They significantly impact the overall coupling coordination between the two systems, making them a critical factor in advancing the digital economy. Compared to other factors, they exhibit a neighboring relationship, highlighting their substantial impact on the development and interaction of the two systems. During the three years, there were fluctuations in the value added by secondary and tertiary industries, GDP per capita, and population density. The P-value of GDP per capita and population density followed a "U"-shaped change, decreasing initially and then increasing, while the proportion of value added of secondary and tertiary industries showed an inverted "U" type of change, increasing initially and then decreasing. The observed patterns indicate a threshold effect in how these factors impact the coupling coordination of digital economic development and regional economic resilience. However, with these factors’ continued accumulation and role, the original thresholds are broken, and they begin to affect how the two systems are coupled and coordinated significantly. The change in P-values reflects the ability to transform the industrial structure. In the pre-transformation period, these factors had a more significant coordinating effect on the two systems, indicating the importance of industrial structure transformation. As the structural transformation progresses, the degree of influence on the two systems weakens, leading to changes in the P-values. This suggests that the coordinating effect on the two systems is greater during the pre-transformation period, while its influence becomes less significant as the structural transformation continues.

## 7. Discussion

The emergence of the digital economy as a new economic form injects vitality into contemporary socio-economic development and inevitably exerts a certain influence on the development of economic resilience. Consequently, current research predominantly focuses on the unilateral impact of the digital economy on economic resilience, with limited consideration of how the two systems develop in coordination, overlooking their mutual interdependence. Therefore, this study adopts a new perspective on the collaborative development of the digital economy and regional economic resilience. Firstly, we employ the coupling coordination model for measurement and construct a new comprehensive evaluation system for the two systems. We apply the entropy weight-TOPSIS method for weighted evaluation. Additionally, we utilize data from China’s 31 provinces and municipalities from 2011 to 2020 as research samples. Furthermore, to thoroughly explore the evolution and distribution of the collaborative relationship between the digital economy and regional economic resilience, we conduct discussions on temporal-spatial distributions. After dividing the regions, we also observe the differences in coupling coordination effects under regional agglomeration effects. We further employ spatial autocorrelation analysis to observe adjacent effects and leap features during the research period. Finally, we use a geographic detector to investigate the driving factors of the collaborative impacts between the two systems. The research findings provide a basis for the collaborative development and joint governance of the digital economy and regional economic resilience.

Through the comprehensive evaluation system constructed in this research period, the Development Index of China’s digital economy and the Comprehensive Development Index of regional economy resilience exhibit an annual upward trend. However, the Comprehensive Digital Economy Index demonstrates a "leapfrog" increase, while the Regional Economic Resilience Index undergoes a "steady-state" transformation. This aligns closely with the conclusions drawn by Zhang et al. (2017) [54] and Wang et al. (2023) [55]. While these perspectives have delved further into regional disparities in China’s digital economy and regional economic resilience as individual systems, this study focuses on the synergistic development between the digital economy and regional economic resilience. Zhang et al. posit that China’s digital economy is generally in a state of growth but faces certain imbalances. Wang et al. believe that China’s regional economic resilience is experiencing an overall improvement, though a step-like pattern exists in the east-west direction.

Furthermore, this study observes that during the research period, the coupling coordination between the development of China’s digital economy and the regional economic resilience demonstrates a positive performance, showing continuous improvement. However, there exists a disparity in the level of coupling coordination between regions, manifesting as a step-like distribution of "higher in the east and lower in the west." In conjunction with the findings of Zhang et al. (2017) [54] and Wang et al. (2023) [55], we note that the distribution characteristics of the synergistic development of the digital economy and regional economic resilience exhibit similarities with the distribution characteristics of individual systems, both displaying a certain degree of imbalance, with the step-like distribution pattern persisting. Additionally, this paper divides the observed economic comprehensive areas into levels of aggregation disparities, revealing a hierarchical presentation of coupling coordination. The first level encompasses the eastern, northern, and southern coastal regions, constituting the leading areas. The second level comprises the middle reaches of the Yangtze River, the northeastern region, the middle reaches of the Yellow River, and the southwestern region, revolving around the national average level, representing the average areas. The third level encompasses the northwestern region, where the coupling development level of the two systems lags.

In Li et al.’s (2021) study on the digital economic system [56], conclusions were drawn regarding the distribution disparities among China’s eight major comprehensive economic zones. It was found that the digital economy primarily concentrates on coastal areas, the Yangtze River’s middle reaches, and the Yellow River’s middle reaches. This aligns with the performance of the synergistic coupling between the digital economy and regional economic resilience discussed in this paper. Additionally, Li et al. observed a significant positive spatial correlation in the level of digital economic development, which is consistent with the spatial correlation findings in this study. Under different regional transitions, past research has suggested that the digital economy offers greater possibilities and flexibility for regional economic development, resulting in a marginal increment effect and a penetrative solid influence on enhancing resilience. These aspects can closely integrate with the region’s ability to continuously adjust in the face of external environmental changes, thereby maintaining overall economic stability [4,51]. Developed regions find it easier to embrace the digital economy’s new ideas and technologies, facilitating collaborative adjustments in economic structure and enhancing resilience against risks [57]. Consequently, the digital economy and regional economic resilience exhibit a more pronounced synergistic effect. Furthermore, this study conducts a spatial correlation analysis to validate the research samples’ coupling coordination. It is observed that there is a spill-over effect in coordination between neighboring areas, but the patterns triggering leaps exhibit variations. However, for the most part, a spatially stable pattern is characterized by " Local migration," indicating that a lag by the spill-over effect from adjacent areas influences different regional disparities.

Moreover, according to the results obtained from the geographical detector analysis, all variables positively affect the coupling coordination level of the two systems. Notably, online payment capability exerts the most significant influence. Research by Du et al. (2023) [29] and Song et al. (2022) [58] has previously indicated that the digital economy demonstrates unique elasticity, with consumer spending levels being able to promote the impact of the digital economy on economic resilience. Furthermore, this is correlated with improving people’s online payment habits. Among other findings, the impact of fiber optic cable length is relatively high, while per capita GDP and population density have lower and fluctuating influences. Li et al.’s (2021) [56] research revealed that material input has the most significant impact on digital economic development, and investment in labor costs is crucial to digital economic growth. However, based on the conclusions drawn in this paper, elements of a similar nature, such as fiber optic cable length and population density, exhibit disparities in their impact on the performance of both the digital economy and regional economic resilience systems. Additionally, the level of external cooperation plays a critical role in the synergistic development of the digital economy and regional economic resilience while the influence of industrial structure fluctuates.

The findings of this study provide a foundation for understanding the collaborative development of the digital economy and regional economic resilience. The comprehensive evaluation system established during the analytical process for the digital economy and regional economic resilience has meaningful implications for research in other regions worldwide. Furthermore, the clear research conclusions on the evolutionary patterns and distribution characteristics of the collaborative development of the two systems, obtained through coupling coordination and spatial autocorrelation, offer valuable references for the joint governance of the digital economy and economic resilience in the sampled regions. These findings also hold illuminating potential for areas beyond the sample. In light of the heterogeneity in the synergistic state of China’s digital economy and regional economic resilience identified in the conclusions, targeted development strategies can be formulated for vulnerable regions, drawing reference from leading regions. This approach can facilitate synchronized progress in the digital economy and regional economic resilience, efficiently harnessing synergistic effects.

At the same time, this study has certain limitations. Firstly, the research sample in this paper consists of Chinese provincial-level units, leaving ample room for expanding the research data. Future studies could focus on more granular regions, such as urban clusters and county-level areas, which would be conducive to discovering the collaborative development patterns of the digital economy and economic resilience within regions and proposing targeted governance recommendations. Additionally, it would be beneficial to conduct separate discussions on regions with different development statuses and categorize discussions based on the characteristics of regional industrial development. This would allow for a concentrated examination of the collaborative development characteristics of the digital economy and economic resilience under the dominance of different types of industries. Finally, there is significant room for exploration regarding the factors influencing the collaborative development of the digital economy and regional economic resilience. Critical factors for the collaborative governance of the digital economy and regional economic resilience from multidimensional and multi-perspective angles await further investigation. The precise identification of favorable development factors as regulatory targets can be a focal point for future research.

## 8 Conclusion and recommendations

### 8.1 Conclusion

Based on the analysis conducted, the following conclusions have been drawn:

China’s digital economy development and regional economic resilience of the comprehensive development level between 2011 and 2020 showed an upward trend year by year. Still, the regional economic resilience composite index showed a "smooth" change, while the digital economy composite index showed a "leaping" rise.

Throughout the study period, there was a strong coupling between the development of China’s digital economy and the resilience of regional economies, leading to continuous progress. However, there was a noticeable disparity in the level of inter-regional coupling coordination, manifesting as a "high east, low west" ladder-type distribution. The economic integration zones demonstrated hierarchical levels of agglomeration. The first level comprised the eastern, northern, and southern coastal regions, which were the leading regions. The second level comprises average regions closer to the national average level. These regions include the middle reaches of the Yangtze River’s northeastern region and the middle reaches of the Yellow River. The northwestern and southwestern regions, classified as backward regions due to the lagging coupling coordination level of the two systems, fall under the third level. By utilizing the kernel density estimation method, a widening gap in the coupling coordination level between the eight economically integrated regions was observed, suggesting a polarization trend.

Spatial analysis reveals that the coupling coordination level among China’s 31 provinces and municipalities exhibits a positive spatial correlation, displaying clear spatial agglomeration patterns. The Moran’s I index follows a "U"-shaped distribution, initially decreasing and increasing, indicating a shift from low to high agglomeration levels. The local Moran’s scatter plot indicates a prevalent pattern of local migration, with most provinces and municipalities in the first and third quadrants. This suggests a spatial stabilization pattern among the regions. Furthermore, upstream migration is observed in Liaoning, Jilin, Heilongjiang, and Henan provinces, while downstream migration occurs in Hainan province.

The results of the Geodetector analysis indicate a positive influence of each variable on the coupling coordination level of the two systems. Among them, the online payment capacity has the most significant effect. Moreover, the coupling and coordination level is more significantly influenced by the total import and export amount of foreign-funded enterprises, online payment capacity, and length of fiber-optic cables. On the other hand, variables such as GDP per capita, population density, and the proportion of value added in the secondary and tertiary industries have a lower impact intensity and are more susceptible to fluctuations.

### 8.2 Recommendations

Based on the aforementioned conclusions, it is evident that China’s digital economic development and regional economic resilience are experiencing orderly enhancements, and their coupling role has also been strengthened. However, regional disparities in development levels persist and are likely to widen further. The development trends of these two systems are challenging to synchronize due to the influence of different economic environments.

In light of this, this study makes the case that the digital economy and regional economic resilience have a significant synergistic relationship. As the development of one system progresses, it is essential to consider the role and mechanisms of the other system. Cities in the eastern, northern, and southern coastal areas have demonstrated excellent performance in both systems and can effectively combine and leverage their advantages. These cities should focus on resource utilization within their regional engine area while gradually establishing a leading role. They can strengthen inter-regional development and cooperation by leveraging their coastal geographical locations.

Furthermore, by strengthening inter-regional cooperation and establishing a complementary and mutually beneficial pattern, these coastal cities can fully unleash their spillover effects, thereby radiating their influence to other economic zones. East-West nexus regions should focus on upgrading their industrial structure and efficiently harnessing the high-quality resources and skills that overflow from the coastal region. They can break through the barriers to their development by aligning with strategic initiatives such as "the rise of the central part of the country," the Belt and Road Initiative, and the Yellow River Basin’s excellent development. These regions can serve as crucial hubs for exchanges and communication between the East and the West.

Given the relatively underdeveloped state of the Northwest and Southwest regions, it is challenging to bring about rapid changes in the lagging development of the two systems. Formulating development policies that align with the local conditions and regional development policies is necessary. The government should prioritize advancing the development of favorable industries and creating appropriate initiatives to entice businesses. In the context of the national development of the digital economy and the creation of new economic models, this will facilitate the introduction and development of industries with unique characteristics, aligning with the development of their economic resilience. The government should prioritize developing advantageous industries in the area, and customized strategies for introducing new businesses should be created.

## Supporting information

S1 Dataset (XLSX)
